# Comparison between dietary, parenteral, and genetic iron overload on bone health reveals secondary iron overload as a driver of cortical bone loss and fracture risk in mice

**DOI:** 10.1093/jbmrpl/ziaf118

**Published:** 2025-07-13

**Authors:** Peter Steele-Perkins, Dilay Yilmaz, Yves Walther, Alessa Wagner, Rossana Paganoni, Martina Rauner, Ulrike Baschant, Maja Vujić Spasić

**Affiliations:** Institute of Comparative Molecular Endocrinology, Ulm University, 89081 Ulm, Germany; Institute of Comparative Molecular Endocrinology, Ulm University, 89081 Ulm, Germany; Institute of Comparative Molecular Endocrinology, Ulm University, 89081 Ulm, Germany; Institute of Comparative Molecular Endocrinology, Ulm University, 89081 Ulm, Germany; Institute of Comparative Molecular Endocrinology, Ulm University, 89081 Ulm, Germany; Department of Medicine III and Center for Healthy Aging, Medical Faculty, University Hospital Carl Gustav Carus, Dresden University of Technology, 01307 Dresden, Germany; Department of Medicine III and Center for Healthy Aging, Medical Faculty, University Hospital Carl Gustav Carus, Dresden University of Technology, 01307 Dresden, Germany; Institute of Comparative Molecular Endocrinology, Ulm University, 89081 Ulm, Germany

**Keywords:** HFE-hemochromatosis, cortical bone, fracture, osteoporosis, parenteral, nutritional iron, genetic iron, liver

## Abstract

More than 18% of the global population suffers from osteoporosis and its associated fracture risk each year. Among the many factors implicated in osteoporosis development, high iron levels have been implicated in bone loss in mice and patients. Here, we performed a comparative analysis of the effect of iron overload induced via diet, injections, or genetic factors, on overall bone health and mechanical bone strength. We used female mice, given the higher risk of osteoporosis and associated fractures in women than in men. We show that dietary iron overload induced trabecular remodeling in the spine but not in the femur, with potentially pre-pathogenic structural changes. By contrast, iron injections caused severe bone deficits across all sites measured. Interestingly, the loss of cortical bone emerged as a common hallmark of secondary iron overload and was associated with decreased mechanical strength in mice. However, no bone anomalies were observed in mice with genetic iron overload, demonstrating that iron overload per se does not suffice to induce bone loss in genetic hemochromatosis. Collectively, our study shows that iron overload-induced by diet and injections, but not genetically, induces selective and specific bone deficits, which are associated with decreased bone mechanical strength in mice.

## Introduction

Iron is an essential micronutrient required for a myriad of biochemical and cellular processes. Maintaining iron levels within a narrow range is critical for overall health. The control of circulating iron is achieved by factors that sense iron levels and subsequently regulate the expression of iron-related genes and proteins. One of the central regulatory mechanisms is the hepcidin-ferroportin axis. Hepcidin is the iron hormone whose levels increase upon iron loading and decrease when insufficient iron is present in the system.^1–3^ Molecularly, hepcidin acts by binding to iron exporter ferroportin, causing its degradation and iron retention within the cells.^4^^,^^5^ Insufficient levels of iron lead to iron deficiency, anemia, and impaired erythropoiesis, while iron excess, due to either genetic factors or excessive dietary or parenteral iron intake, is implicated in a range of pathological conditions, leading to multiple organ failure/damage, and increased mortality.^6–8^

One organ that appears particularly vulnerable to iron fluctuations is the bone.^9^ Bone is a dynamic tissue that undergoes remodeling via coordinated action of bone cells, including osteoblast, osteoclast, and osteocytes, which mediate bone adaptation to micro-environmental and systemic changes.^10^^,^^11^ Iron and a well-rounded, nutrient-rich diet are required to maintain optimal bone health.^12^ Iron overload affects bone remodeling by suppressing bone formation and accelerating bone resorption. It does so by modulating the cellular capacity of bone cells: it inhibits osteoblast differentiation and mineralization,^13^ promotes osteocyte apoptosis,^14^ along with osteoclast differentiation and activity.^13^^,^^15^ For example, iron-sulfate treatment inhibited osteogenic differentiation of bone marrow mesenchymal stem cells without influencing adipogenic and chondrogenic differentiation,^16^ and negatively impacted the activity and extracellular matrix mineralization of mature osteoblasts.^17^ By contrast, ferric iron promoted osteoclast differentiation and osteocyte apoptosis through the production of reactive oxygen species (ROS).^14^^,^^15^

At systemic levels, the effects of dietary iron overload on bone status were dependent on the timing of the diet and on the genetic background of mice.^18^ For example, male mice on an SV129 genetic background developed significant bone loss during the short-term (6 wk) treatment, whereas C57BL/6 mice were mostly unresponsive.^18^^,^^19^ In patients, however, clinical data showed that increased total body iron stores could be an independent risk factor for accelerated bone loss, even in healthy individuals.^20^ On the other hand, a moderate increase in dietary iron consumption, particularly in women who show a significantly increased risk for bone loss, may be advantageous to prevent bone loss and associated fractures.^21^ In contrast to dietary iron, excessive iron loading achieved by iron injections has been repeatedly associated with increased osteoporosis, fractures, cortical thinning, lower bone mass, and bone density in mice,^22^ and patients.^23^^,^^24^ In particular, patients with congenital or acquired anemias who require regular blood transfusion therapies to increase systemic iron levels show an overall increased risk of osteoporosis and fractures.^25^ Similar bone pathologies, including an increased occurrence of osteoporosis, osteoarthritis, arthropathy, joint replacements, and a consecutive decrease in BMD, were reported in HFE-hemochromatosis patients,^6^^,^^26^^,^^27^ who suffer from genetic iron overload due to the disruption of the iron sensing-hepcidin pathway.^28–30^ However, studies in mice with constitutive and tissue-specific *Hfe* deficiency^31^ and recent patient data from the UK,^32^ demonstrated that the iron levels in HFE-HH are insufficient to induce bone loss under steady-state conditions, contrasting initial reports.^33–35^

However, there is a disparity between secondary iron overload conditions and primary (genetic) in the molecular mechanisms of iron origin, the pattern of iron loading, the type of iron, as well as in the magnitude and temporal span of iron loading, all of which exert profoundly different effects on bone and overall systemic health. Thus, we designed our study to systematically and comparatively investigate the effect of chronic iron overload, caused by diet, parenteral iron intake, and genetic hemochromatosis, on overall bone health and strength. Moreover, we used female mice in particular, given that the prevalence of osteoporosis and osteoporosis-related fractures is twice as high in women compared to men.^36^

## Materials and methods

### Mice

*Hfe*  ^−/−^ mice and their respective WT littermate controls (further abbreviated as KO and Wt, respectively), were all maintained on C57Bl/6J genetic background. Female mice were used in this study mice were housed under a 12 h light/dark cycle, in a temperature-controlled environment with abundant enrichment. Mice were maintained under SPF conditions. The mice had ad libitum access to water and standard chow Ssniff V1534 containing 189 mg of iron per kg (Snniff). The mice were then randomly assigned to a control or treatment group.

#### Iron-rich diet (IRD)

**Figure 1:**
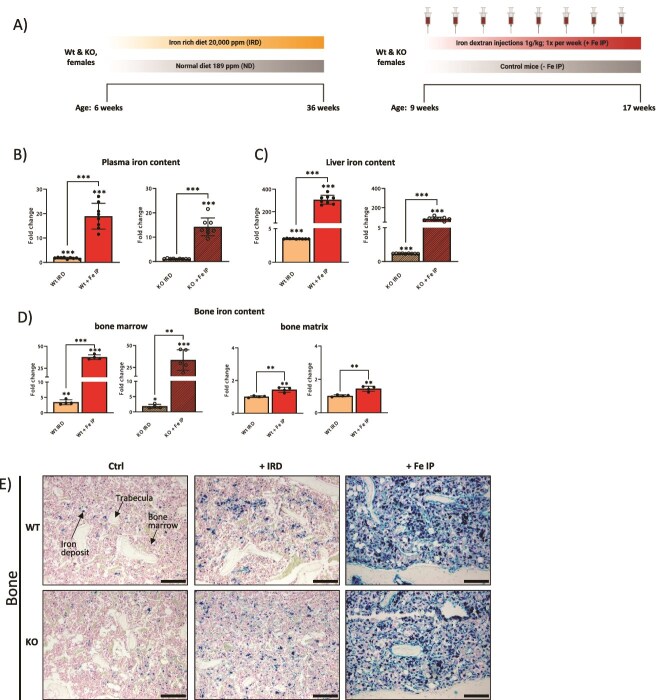
Effects of dietary and parenteral iron overload on systemic iron levels in Wt and KO mice. (A) Schematic overview of the experimental set-up for iron-rich diet (IRD) and intraperitoneal iron (Fe IP) injections, with their respective controls and timelines. (B-D) Comparison of plasma iron, liver iron, and bone iron levels in Wt and KO mice upon IRD and Fe IP expression. Data are shown as fold change calculated relative to value in untreated mice (set to 1), with significance compared to respective untreated mice indicated above the column. Bone iron content is measured within bone marrow and bone matrix. (E) Iron deposition in the tissues was visualized using Prussian blue staining. Scale bar indicates 50 mm. Arrows indicate iron deposits, trabeculae, and bone marrow. The data is presented as the mean ± SD, with each symbol representing an individual animal. ^*^p < .05, ^*^^*^p < .01, ^*^^*^^*^p < .001.

At the age of 6 wk, mice were placed on a Ssniff standard chow V1534 or the same diet supplemented with 2% carbonyl iron (20 000 mg carbonyl iron/kg) for 30 wk (Figure 1A).

#### Iron-injections (Fe IP)

At the age of 9 wk, mice were injected once per week with low molecular weight iron dextran D8517 (Merck) at 1 g/kg body weight, i.p., for 8 wk in total (Figure 1A).

During the course of the experiments, mice were monitored and scored for potential changes in body weight, tumor development, body temperature, behavioral changes, mobility, respiration, and general condition. At the end of the experiments, mice were euthanized and tissues were collected, according to the University Animal Care Committee.

### Ethics statement

Animal experiments were approved and performed in accordance with the University Animal Care Committee and the Federal Authorities for Animal Research (RPT0350-9185-12/10/2/1565) (Regierungspräsidium Tübingen). All authors complied with the guidelines for animal research: reporting of in vivo experiments (ARRIVE).

### Micro-CT

Femurs, tibias and vertebrae (L5) were collected from the mice and fixed in 4% formaldehyde for 3 d before being stored in 0.5% formaldehyde. Three-dimensional micro-CT (μCT) analysis was performed on the isolated bones using SkyScan 1176 vivo high-resolution μCT (Bruker). Bones were scanned using a 9 μm pixel size, with a 0.5-1.0° rotation step, and a 0.5 mm aluminum filter employed. Reconstruction of sample images was carried out using the N-Recon program; all samples were assigned a uniform dynamic image range with a minimal value (attenuation coefficient) of 0 and a maximal value (attenuation coefficient) of 0.1. All samples were then orientated using the data viewer program supplied with the machine.

For analysis of the trabecular within the femoral metaphysis, a reference point within the growth plate was selected, and then an offset of 350 slices with a height of 300 slices (2.64 mm) was chosen for each bone. Manual contouring was employed to isolate trabecular bone from cortical bone. Femoral cortical bone was analyzed mid-shaft with an offset of 700 slices and a height of 100 slices (0.88 mm), again selected with manual contouring. For L5 vertebrate analysis, the reference point was generated using the edges of the vertebrate, with a 200-slice (1.76 mm) region of interest (ROI) generated in the center. Manual contouring was used for the isolation of the trabecular for analysis.

These defined ROIs were then used for analysis using the CTAn analysis program, with morphological parameters for assessing trabecular status, such as bone volume/tissue volume (BV/TV), trabecular thickness (Tb.Th), trabecular separation (Tb.Sp), and trabecular number (Tb.N), and for cortical status, cross-sectional thickness (Cs.Th) and mean cross-sectional bone area. All analyses were performed according to the guidelines issued by the ASBMR Histomorphometry Nomenclature Committee.^37^ Additionally, CTAn was used for the analysis of BMD. Models of the ROI were generated using the CTVox program provided by SkyScan.

For further trabecular structural analysis to determine the plates-to-rod composition within vertebrae, the BoneJ plugin for ImageJ (Version 1.54f, National Institutes of Health) was used. Utilizing the 3D models created from the ROIs in the previous analysis, each model was analyzed for the largest possible ellipsoid that fits in each given volume resulting in the ellipsoid factor (EF).^38^ These ellipsoids were used to classify plate-like, rod-like structures, or intermediates. The 3D renders were created using the Skeletonize3D plugin in ImageJ. Modeling was run 6 times per sample, with the average taken to minimize any variability.

### Bone histomorphometry

Static histomorphometry was carried out on decalcified femurs from the mice. After μCT analysis, bones were decalcified in 15% EDTA (changed every 3 d) for 14 d on a rocking plate. The bones were dehydrated through an ethanol series, then embedded in paraffin and sliced into 10 μm sections. Sections were deparaffinized and stained for tartrate-resistant acid phosphatase (TRAP), and counter-stained with hematoxylin. Analysis of osteoblast number and area, osteoclast number and area, and osteocyte number was performed using the Osteomeasure Analysis system (Osteometrics). Images of TRAP staining were produced with a microscope (Olympus) at 20× magnification.

### ELISA

Bone turnover markers indicating osteoclast activity via CTX-1, and osteoblast activity via P1NP, were measured by ELISA according to the manufacturer’s instructions (Immunodiagnostics).

### Hematological parameters

To assess hematological parameters, blood was collected in an EDTA-coated tube and analyzed using a Hematology Analyzer (Scil Animal Care Company GmbH). Blood was then centrifuged at 2300 g for 12 min, and plasma was collected and stored at −80°.

### Iron measurement and staining

Blood was collected into heparinized tubes (in case of IRD treatments) or EDTA tubes (in case of Fe IP treatments) and plasma separated after centrifugation at 1500 × *g* for 12 min. Plasma iron and the non-heme iron in the liver were analyzed using a modified method as previously described.^28^^,^^39^ Bone and liver iron deposition was visualized using Prussian blue staining. Bone sections were prepared as above, and liver sections were cut from paraffin-embedded tissue previously fixed in 4% paraformaldehyde (PFA). These sections were stained for iron using 1% HCl and 2% potassium hexacyanoferrate II trihydrate (Merck) for 60 min. Nuclear Fast Red solution was used as a counterstain for 10 min (Sigma-Aldrich). Images were taken with a microscope (Olympus) at 20× magnification.

Bone iron content in the bone marrow and bone matrix was quantified from Prussian blue-stained bone sections. For each section, 5 smaller areas were randomly selected from the marrow or trabeculae for analysis.

This analysis was carried out in CellProfiler 2.4.7. Color images were deconvoluted to convert the blue signal into a greyscale image. This was then further thresholded to remove the background signal, and the mean intensity was measured. The mean of the 5 sites was then used to represent one mouse for statistics, with an animal number of 4-5 per condition.

### Biomechanical testing (3-point bending test)

Three-point bending tests (Zwick Roell) were performed for biomechanical analyses of the tibiae. Paraformaldehyde-fixed tibiae were rehydrated overnight in PBS. For testing, tibiae were placed onto 2 supports with an intermediate distance of 6 mm. The mechanical force was applied vertically in the middle of the tibial midshaft. The measurement started after reaching a preload of 1 N and was performed with a load rate of 0.05 mm/s until failure. Results of the maximal applicable force (Fmax) as an indicator of cortical bone strength were evaluated using testXpert II—V3.7 software (Zwick Roell).

### Statistical analysis

Statistical analysis was performed using GraphPad Prism software (GraphPad Software), with results shown displaying mean plus SD. Normality was tested using the Shapiro–Wilk normality test. For comparison, when the resulting data was normally distributed unpaired Student’s *t*-test was used, and if non-normally distributed, the Mann–Whitney U test was used. Correlation was tested using Pearson correlation coefficients with normally distributed data, and nonparametric Spearman correlation with non-normally distributed data, then further assessed using simple linear regression. Statistically significant differences are indicated as *p* < .05 (^*^), *p* < .01 (^*^^*^), and *p* < .005 (^*^^*^^*^). The ROUT method (1%) was used to identify potential outliers.

## Results

To compare the effects of iron excess on bone microarchitecture and its mechanical strength, we established 3 models of chronic iron overload: (1) a model of dietary iron overload, whereby mice were fed an iron-rich diet (IRD) for 6 mo, (2) a model of parenteral iron overload, achieved by i.p. injections of 1 g/kg iron dextran once per week for 8 wk in total (short: Fe IP), and (3) a model of genetic iron overload, where *Hfe* KO mice were either untreated or received an additional surplus of exogenous iron in form of IRD or Fe IP (Figure 1A). In all cases, the mice tolerated the iron administration well, with no abnormal behavior or ailment. Moreover, all mice in our study were maintained on the same nutrient-rich diet, thereby excluding any deficiency in the dietary formulation as a reason for potential bone deficits.^12^

### Dietary and parenteral iron overload induces heavy, yet distinct iron loading

We first assessed the systemic iron status in all cohorts to identify the degree of iron loading within our models. We show that long-term IRD in Wt mice caused a significant increase in circulating plasma iron levels and increased iron deposition in the liver and the bones, with iron being deposited in the bone marrow (Figure 1B-E, Table 1). Similarly, KO mice showed a statistically significant yet mild increase in liver and bone iron levels when compared to the KO mice on a standard diet (Figure 1C-E, Table 1). In contrast to IRD, parenteral iron loading produced a dramatic increase in iron levels in the blood, liver, and bones and induced a gross iron deposition in tissues (Figure 1B-E). Iron deposits were almost exclusively located in the bone marrow (a minimal fraction was also detected in the bone matrix) (Figure 1D and E). Importantly, Fe IP treatment produced overall similar effects in both genotypes (Figure 1B-E, Table 2). Furthermore, in the blood, IRD induced relatively minor statistically significant changes (Table 1), whereas Fe IP profoundly affected the composition of all blood cells, leading to a decreased amount of red blood cells and hemoglobin, and an increased number of immune cells (Table 2).

**Table 1:** Major iron and blood indices in Wt and KO mice upon iron loading by iron-rich diet (IRD).

**Strain**	**Wt**		**Wt**	**KO**		**KO**
**Treatment**	**ND**		**IRD**	**ND**		**IRD**
***N***	**10**		**9**	**7**		**8**
**Plasma Fe (μg/dL)**	144.7 ± 34.22	^*^^*^^*^	250.8 ± 45.91	214.6 ± 17.46		233.0 ± 38.69
**Liver Fe (μg/g)**	523.6 ± 52.47	^*^^*^^*^	1824 ± 21.35	1542 ± 137.7	^*^^*^^*^	1794 ± 42.85
**Bone Fe (mean int)**	0.021 ± 0.004	^*^^*^	0.072 ± 0.017	0.024 ± 0.004	^*^	0.045 ± 0.015
**RBC (10^6^/mm^3^)**	10.19 ± 0.4558	^*^^*^	9.434 ± 0.4677	9.944 ± 0.2623	^*^	9.36 ± 0.5342
**HGB (g/dL)**	15.14 ± 0.8489		15.43 ± 0.7656	16.49 ± 0.5451		15.81 ± 0.7331
**MCV (μm^3^)**	66.9 ± 1.287		71.56 ± 1.333	71.71 ± 0.7559		74.38 ± 2.066
**HCT (%)**	67.94 ± 2.316		67.43 ± 2.962	71.26 ± 1.708		69.41 ± 2.911
**RDW (g/dL)**	13.56 ± 0.2982		13.56 ± 0.2981	13.18 ± 0.305		13.71 ± 0.1704
**WBC (10^3^/mm^3^)**	7.129 ± 0.8264		8.030 ± 1.237	7.706 ± 0.885		6.94 ± 1.368
**LYM (10^3^/mm^3^)**	5.72 ± 0.6844		6.067 ± 1.059	6.143 ± 0.7458		5.138 ± 1.221
**MON (10^3^/mm^3^)**	0.31 ± 0.07379	^*^^*^	0.4778 ± 0.1093	0.3429 ± 0.0534		0.4 ± 0.1414
**GRA (10^3^/mm^3^)**	1.09 ± 0.2025	^*^^*^	1.489 ± 0.2088	1.229 ± 0.1976		1.4 ± 0.444

Normality was tested using the Shapiro–Wilk normality test. For comparison when the resulting data was normally distributed unpaired Student’s *t*-test was used, and if non-normally distributed, the Mann–Whitney U test was used. The ROUT method (1%) was used to identify potential outliers. Statistically significant differences are indicated as *p* < .05 (^*^), *p* < .01 (^*^^*^), and *p* < .005 (^*^^*^^*^).

Abbreviations: Bone Fe, bone iron content (units mean intensity); GRA, granulocytes; HCT, hematocrit; HGB, hemoglobin; Liver Fe, liver iron content; LYM, lymphocytes; MCV, mean corpuscular volume; MON, monocytes; Plasma Fe, plasma iron content; RBC, red blood cells; RDW, red blood cell distribution width; WBC, white blood cells.

**Table 2:** Major iron and blood indices in Wt and KO mice upon iron loading by iron injection (Fe IP).

**Strain**	**Wt**		**Wt**	**KO**		**KO**
**Treatment**	**−Fe IP**		**+Fe IP**	**−Fe IP**		**+Fe IP**
***N***	**7**		**8**	**7**		**8**
**Plasma Fe (μg/dL)**	54.16 ± 20	^*^^*^^*^	1025 ± 286.5	70.88 ± 17	^*^^*^^*^	1006 ± 259.4
**Liver Fe (mg/g)**	450.6 ± 57.37	^*^^*^^*^	138 222 ± 17 867	1245 ± 71.61	^*^^*^^*^	105 635 ± 23 394
**Bone Fe (mean int)**	0.005 ± 0.003	^*^^*^	0.328 ± 0.121	0.008 ± 0.008	^*^^*^^*^	0.268 ± 0.093
**RBC (10^6^/mm^3^)**	10.92 ± 0.6133	^*^^*^	9.018 ± 0.766	10.37 ± 0.4449	^*^^*^	9.059 ± 0.6322
**HGB (g/dL)**	17.42 ± 1.105	^*^	15.22 ± 1.772	16.72 ± 0.9839	^*^^*^^*^	14.45 ± 0.6821
**MCV (μm^3^)**	60.43 ± 1.272	^*^^*^^*^	64.88 ± 2.416	47.83 ± 1.472	^*^^*^	45.43 ± 0.7868
**HCT (%)**	66.57 ± 4.466	^*^^*^	58.26 ± 7.023	49.9 ± 2.603	^*^^*^	42.04 ± 1.504
**RDW (g/dL)**	13.66 ± 0.2174	^*^^*^^*^	15.24 ± 0.7085	13.94 ± 0.2281	^*^^*^	14.91 ± 0.2485
**WBC (10^3^/mm^3^)**	10.86 ± 1.638	^*^^*^^*^	39.87 ± 7.874	11.29 ± 3.504	^*^^*^^*^	33.24 ± 2.834
**LYM (10^3^/mm^3^)**	8.943 ± 1.365	^*^^*^^*^	23.05 ± 4.751	7.083 ± 3.153	^*^^*^^*^	21.21 ± 2.272
**MON (10^3^/mm^3^)**	0.3571 ± 0.053	^*^^*^^*^	3.975 ± 0.8294	0.44 ± 0.1517	^*^^*^	2.95 ± 0.7171
**GRA (10^3^/mm^3^)**	1.571 ± 0.2498	^*^^*^^*^	12.84 ± 2.841	2.3 ± 1.577	^*^^*^^*^	9.929 ± 1.725

Normality was tested using the Shapiro–Wilk normality test. For comparison when the resulting data was normally distributed unpaired Student’s *t*-test was used, and if non-normally distributed, the Mann–Whitney U test was used. The ROUT method (1%) was used to identify potential outliers. Statistically significant differences are indicated as *p* < .05 (^*^), *p* < .01 (^*^^*^), and *p* < .005 (^*^^*^^*^).

Abbreviations: Bone Fe, bone iron content (units mean intensity); GRA, granulocytes; HCT, hematocrit; HGB, hemoglobin; Liver Fe, liver iron content; LYM, lymphocytes; MCV, mean corpuscular volume; MON, monocytes; Plasma Fe, plasma iron content; RBC, red blood cells; RDW, red blood cell distribution width; WBC, white blood cells.

Collectively, this data shows that iron loading was successfully achieved in mice following both IRD and Fe IP and that significantly heavier and more unconstrained iron deposition was detected in mice receiving Fe IP than in the IRD model.

### Dietary iron overload induces vertebrae remodeling and microstructural changes, indicative of a plate-to-rods phenotype

We first performed μCT analysis of the L5 vertebrae from Wt and KO mice, upon IRD treatment and iron injections (Fe IP) and compared to the bone status of control mice.

**Figure 2:**
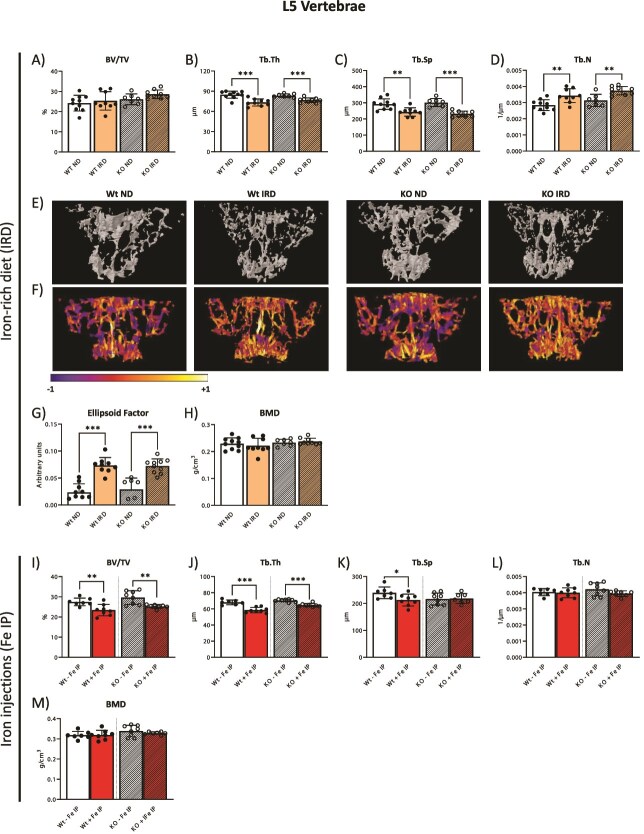
Effects of dietary and parenteral iron overload on the spine. (A-D) Trabecular bone parameters in the L5-vertebra of mice on IRD and controls (ND) were assessed using μCT, displaying bone volume per total volume (BV/TV), trabecular thickness (Tb.Th), trabecular separation (Tb.Sp), and trabecular number (Tb.N). (E) Representative 3D rendering of the L5 trabecular of Wt and KO mice on IRD and controls (ND) in the ROI analyzed. (F) Ellipsoid factor heat maps were generated from the L5 vertebrae trabecular of mice on IRD and controls (ND), with purple indicating a plate-like trabecular structure and yellow a rod-like trabecular structure. (G) Ellipsoid factor indicating trabecular plate-to-rod status was assessed on L5 vertebrae of mice on IRD and controls (ND). (H) Vertebrae L5 BMD of mice on IRD and controls (ND). (I-L) Trabecular bone parameters in the L5-vertebra of mice following Fe IP and controls were assessed using μCT, displaying BV/TV, Tb.Th, Tb.Sp, and Tb.N, respectively. (M) Vertebrae L5 BMD of mice on Fe IP and controls. The data is presented as the mean ± SD, with each symbol representing an individual animal. ^*^p < .05, ^*^^*^p < .01, ^*^^*^^*^p < .001.

Within the spine, we measured no statistically significant changes in the bone volume (BV/TV) parameter of Wt and KO mice maintained on IRD (Figure 2A). However, a decreased Tb.Th leading and an increased number of thinner trabeculae were detected upon IRD in both Wt and KO mice (Figure 2B-D). This data is indicative of trabecular remodeling and microstructural changes induced by IRD within the vertebrae, which was further reinforced in the accompanying 3D model of the ROI, which shows no overall bone volume loss but significant structural alterations (Figure 2E). The microstructural alterations were further investigated using the ellipsoid factor, which allows a classification of trabecular structures as more rod-like or plate-like, with a perfect plate structure giving a value of −1 and a perfect rod 1,^38^^,^^40^ with values being the average value of all the segments analyzed within a ROI. This analysis revealed a distinct shift to a more rod-like trabecular structure in both Wt and KO mice on an IRD (Figure 2F and G), being indicative of a lower bone quality. Importantly, these structural changes were evident only upon chronic dietary iron treatment and occurred both in Wt and KO mice. Despite the observed structural aberrations raised by the IRD, the BMD remained consistent throughout all conditions (Figure 2H).

We next inspected the effect of genetic iron overload on the integrity of the vertebrae. As shown in Figure 2A-H, the presence of genetic iron overload alone did not associate with trabecular remodeling within the spine of KO mice when compared to Wt mice, both maintained on a standard diet. Interestingly, when comparing the effects of dietary iron overload in Wt mice (Wt, IRD) to genetic (KO ND), we measured an increased number of trabeculae, with a decreased Tb.Th and separation in the spine, which associated with significant microstructural changes estimated using the ellipsoid factor (Figure S1A and B), despite the presence of high levels of systemic iron in these mice (Table 1).

In contrast to the effects of IRD, a significant decrease in bone volume was seen in Wt and KO mice following Fe IP treatment, with an accompanying thinning of trabeculae (Figure 2I and J). Curiously, Tb.Sp was significantly decreased in Wt mice but not in KO, and no changes were measured in Tb.N (Figure 2K and L). The accompanying 3D model shows overall bone volume loss, whereas no significant microstructural changes were identified following the implementation of the ellipsoid factor analysis (Figure S2A-C). Moreover, BMD remained unaffected upon Fe IP, recapitulating the effects of IRD (Figure 2M).

Based on these data, we conclude that dietary iron loading is associated with significant microstructural alterations of the spine, contrasting the effects of genetic and parenteral iron overload.

### Parenteral iron overload induces trabecular bone loss

**Figure 3:**
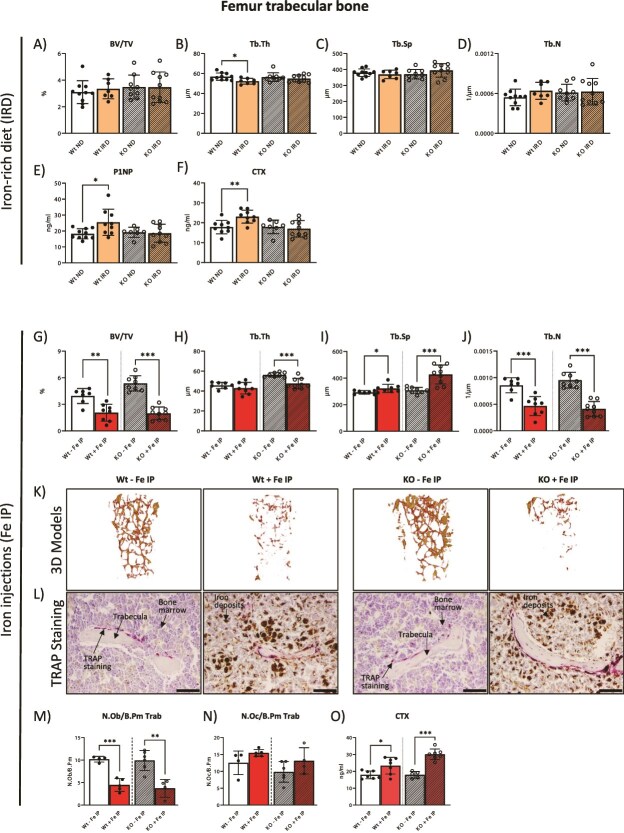
Dietary iron overload does not affect femoral trabecular bone, while iron injections induce gross bone deficits. (A-D) Trabecular bone parameters in the femur of mice on IRD and controls (ND) were assessed using μCT, displaying bone volume per total volume (BV/TV), trabecular thickness (Tb.Th), trabecular separation (Tb.Sp), and trabecular number (Tb.N). (E and F) Serum levels of P1NP and CTX-1 of mice on IRD and controls (ND). (G-J) Trabecular bone parameters in the femur of mice subjected to iron injections (Fe IP) and respective controls were assessed using μCT, displaying BV/TV, Tb.Th, Tb.Sp, and Tb.N. (K) 3D models of femoral trabecular structure in Fe IP treated mice and controls from the measured ROI. (L-N) Tartrate-resistant acid phosphatase (TRAP) staining in the femur of Fe IP treated mice and controls, with the respective bone histomorphometry depicting number of osteoblasts/osteoclast relative to the bone parameter (N.Ob/B.Pm, N.Oc/B.Pm). Scale bar indicates 50 mm. Arrows indicate trabeculae, bone marrow, TRAP staining, and iron deposits. (O) Serum levels of CTX-1 in Fe IP treated mice and controls. The data is presented as the mean ± SD, with each symbol representing an individual animal. ^*^p < .05, ^*^^*^p < .01, ^*^^*^^*^p < .001.

Given the effect of an IRD on the spine, we next investigated the structure of femoral bones, particularly at trabecular sites, using the μCT analysis. Interestingly, and in contrast to the effects of IRD on L5 trabecular bone, we measured no significant alterations in the distal femoral trabecular structure in Wt and KO mice on IRD (Figure 3A-D). This was despite the presence of increased iron deposition in the bones, with iron deposition being restricted to the marrow and not present within the trabecular bone (Figure 1E). Tartrate-resistant acid phosphatase staining was additionally performed in femoral sections to assess trabecular bone cell populations, but no changes in the number and/or surface of osteoblasts and osteoclasts were measured (Figure S3). Interestingly, increased plasma levels of P1NP and CTX-1 markers, suggestive of increased bone turnover rate, were measured only in Wt mice on IRD and not in KO mice (Figure 3E and F).

In contrast to IRD, iron injections induced severe femoral trabecular deficits in both Wt and KO mice, measured by a strong reduction in trabecular bone volume, increased Tb.Sp and decreased Tb.N, while Tb.Th only significantly decreased in KO mice (Figure 3G-J). These deficits are visualized in the corresponding ROI 3D models (Figure 3K). Regarding trabecular bone cell populations, Wt and KO mice demonstrated a significant decrease in osteoblast number, suggesting deleterious actions of Fe IP on osteoblasts and a resulting decrease in bone formation (Figure 3L and M). Osteoclast numbers were marginally, yet not statistically increased, however, the osteoclast activity, measured by the plasma levels of CTX-1, was significantly increased in Wt and KO mice, suggesting that enhanced bone resorption is ongoing following iron injections (Figure 3L, N-O).

### Dietary and parenteral iron overload induces cortical bone loss

**Figure 4:**
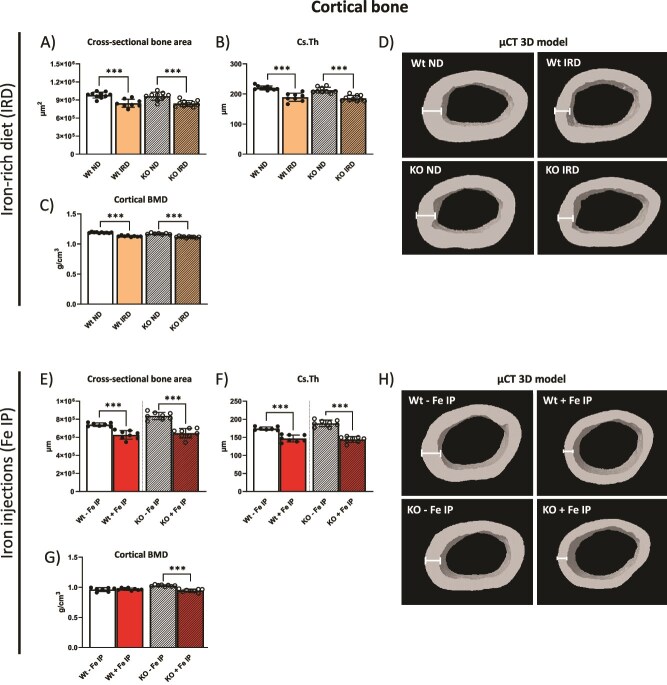
Dietary and parenteral iron overload induce cortical bone loss. (A-C) Cortical bone parameters in the femur of mice on IRD and controls were assessed by μCT, displaying cross-sectional bone area, cross-sectional thickness (Cs.Th) and cortical BMD. (D) Representative 3D rendering of the cortical area of mice on IRD and controls in the ROI analyzed. (E-G) Cortical bone parameters in the femur of mice receiving Fe IP and controls were assessed by μCT, displaying cross-sectional bone area, cross-sectional thickness (Cs.Th.), and cortical BMD. (H) Representative 3D rendering of the cortical area of mice with Fe IP and controls in the ROI. White bars indicate measured cross section of cortical bone. The data is presented as the mean ± SD, with each symbol representing an individual animal. ^*^p < .05, ^*^^*^p < .01, ^*^^*^^*^p < .001.

Lastly, the μCT analysis of the femoral cortical bone was performed. This analysis revealed significant bone loss after IRD in both Wt and KO mice, with a decreased cross-sectional bone area and cortical thickness, and a minimal yet statistically significant decrease in BMD when compared to respective control mice (Figure 4A-C). This is displayed in representative 3D models rendered from the μCT ROI (Figure 4D). While dietary iron overload induced cortical bone loss, the effects of genetic iron overload on cortical bone were not statistically significant (Figure 4A-C; Figure S1C).

Cortical bone was likewise heavily impacted following Fe IP, with significantly decreased bone volume and Cs.Th seen in both KO and Wt mice upon Fe IP (Figure 4E and F), while BMD was only minimally decreased in KO mice (Figure 4G). Cortical bone loss is displayed in representative 3D models rendered from the μCT ROI (Figure 4H). This data shows that the loss of cortical bone is a common target of chronic dietary and parenteral iron overload, contrasting their effects on the trabecular bone.

### Decreased bone mechanical strength is predominant in KO mice

So far, our data shows that long-term dietary iron supplementation, as well as iron injections, result in the thinning of the cortical femoral bone. To investigate whether the observed changes impact overall bone strength, the 3-point bending test was applied to tibia bones, in particular testing the properties of the mid-diaphysis, which is typically governed by cortical status.

**Figure 5:**
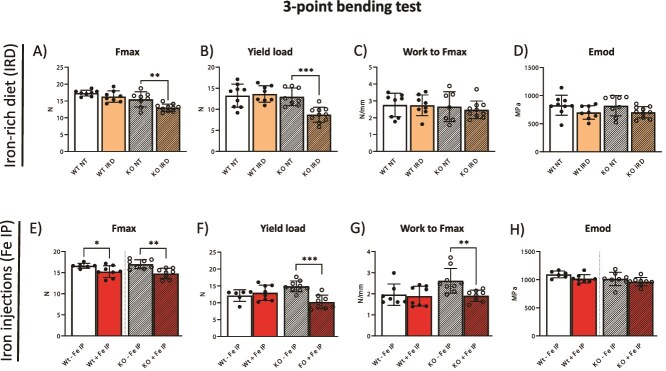
Bone mechanical strength in Wt and KO mice following IRD and Fe IP. (A-D) Mechanical integrity was assessed in tibiae using 3-point bending test with maximal applicable force [N] (Fmax), yield load [N], work-to-fracture [N/mm], and elastic modulus [N/mm^2^ = MPa] (Emod), parameters in mice on IRD and controls. (E-H) Cortical mechanical integrity was also assessed via Fmax, Emod, yield load, and work-to-fracture in mice receiving Fe IP and controls. The ROUT method (1%) was used to identify potential outliers. The data is presented as the mean ± SD, with each symbol representing an individual animal. ^*^p < .05, ^*^^*^p < .01, ^*^^*^^*^p < .001.

Upon IRD, only KO mice showed reduced Fmax, and yield load (which are the measurements of the maximum force required to induce bone failure, and the load at which the bone begins to undergo permanent deformation, respectively), while the amount of energy required to reach Fmax during the bending process remained the same (Figure 5A-C); in contrast to KO mice, Wt mice were not affected (Figure 5A-C). This data indicates a decreased bone mechanical strength in KO mice upon IRD, although dietary iron supplementation induced cortical bone loss in both genotypes (Figure 4). However, no differences regarding elastic modulus (Emod), a measure of recoverable stiffness in bone when force is applied, were measured across both IRD Wt and KO mice (Figure 5D).

Importantly, upon Fe IP, a significant decrease in Fmax value was measured both in WT and KO mice, while yield load and work-to-Fmax were only decreased in the KO mice on FeIP (Figure 5E-G); no change was detected for Emod in any condition (Figure 5H). This data implies that upon force application, the bones of KO mice upon Fe IP are more vulnerable to both permanent damage and fracture, which is also reflected by decreased cortical BMD (Figure 4G).

Collectively, this data shows that the elasticity of the bones is not impacted by either form of iron, while diminished bone mechanical strength prevails in KO mice upon a surplus of exogenous iron.

## Discussion

Osteoporosis is a major public health threat characterized by low bone mass, microarchitectural deterioration of bone, and an increased risk of developing fractures.^41^ Among several factors implicated in osteoporosis development, high iron levels have gained the spotlight due to associative studies reporting different extents of bone loss in iron overload conditions, both in mice and in patients.^9^ Here, we performed a comparative analysis of the effect of chronic iron overload, caused by diet, parenteral, or genetic factors, on overall bone health and mechanical bone strength using female mice, given the higher risk of osteoporosis and associated fractures in women than in men.^36^

We demonstrate that chronic iron overload induces selective and specific bone deficits. More precisely, we show that long-term dietary iron loading (IRD) causes significant trabecular remodeling in the spine, while the femoral trabecular structure was seemingly unaffected. The observed bone deficits were associated with potentially pre-pathogenic structural changes within the vertebrae, and these findings were homogenous between Wt and KO mice. The plate-to-rod phenomenon is important, as transitions in trabecular geometry to the more rod-like form are associated with an increased osteoporosis risk.^42^ Importantly, in the context of patient care, these subtle alterations can be assessed non-invasively using HR-pQCT and can be used in conjunction with BMD (the golden standard of bone health), increasing the accuracy of assessment, or in the absence of a diagnostic BMD reduction, for detecting patients with osteoporosis, secondary osteoporosis, osteopenia, and fracture risk.^43^ Indeed, our mouse models showed no difference in BMD of the vertebra, yet we observed a plates-to-rod phenotype in the L5 vertebra, indicative of lower bone integrity. These alterations may potentially be present in iron-overloaded patients; thus, utilizing these observations could allow for earlier detection and a more effective treatment course in patients with iron overload. In contrast to the effects of IRD, excessive parenteral iron overload induced severe bone loss in Wt and KO mice, with defects seen across all sites measured, supporting previous studies.^22^^,^^23^

Our study further identified the loss of cortical bone as a common target of exogenous iron loading induced by both IRD and Fe IP, being in line with recent data.^12^ However, genetic iron overload, due to lack of HFE, failed to induce any changes in cortical bone, while only in conditions of additional exogenous iron loading is the balance tipped toward bone anomalies: a thinning of the cortical bone exclusively, without any change in trabecular bone volume. These effects are particularly alarming considering that cortical thinning is known to strongly influence susceptibility to fractures, as demonstrated by this and previous studies,^44–46^ which grossly impacts health, and quality of life, even leading to higher mortality rates.^47^^,^^48^

While dietary iron overload without associated factors is relatively rare, there are certain cases where it can occur, for example, from the consumption of food highly rich in iron or due to the use of over-the-counter supplements that include iron,^49–51^ of which an already high global consumption has increased since COVID-19 occurred.^52^ These factors, in combination, are potentially leading to inappropriately high iron loading, especially in the context of aging populations, who are significantly more affected by bone deficits. Another highly vulnerable population to this particular form of iron loading may be asymptomatic HFE patients, who are mostly undiagnosed until the age of 40. In these patients, chronic consumption of dietary iron-rich nutrients could potentially result in a bone phenotype, as documented by our study: a thinning of the cortical bone and increased susceptibility to fractures. Considering how widespread this mutation is, a large population of undiagnosed patients is left vulnerable to preventable bone health disturbances.

Lastly, when addressing the effect of iron overload on bone integrity, several issues must be considered. The levels of systemic iron raised by the dietary iron approach are also highly dependent on the duration and timing of the diet, as well as genetic background and sex.^12^^,^^18^ By contrast, iron injections in mice result in profoundly more iron loading than IRD,^22^^,^^53^^,^^54^ but also the type of iron here is in question: iron injections produce high levels of redox-active iron and ROS, which in turn triggers severe bone loss; these effects may be prevented by the use of antioxidants, DFO, or ferroptosis inhibitors.^22^^,^^55^

Interestingly, and in contrast to dietary and parenteral iron overload, genetic iron overload does not suffice to induce bone loss. Our current, previous and number of recent studies using different hemochromatosis mouse models show that mice lacking *Hfe*,^31^ transferrin receptor 2,^56^ hemojuvelin, and hepatocyte-specific *Alk2* and *Alk3*, do not develop bone loss,^57^ contrary to potential site-specific bone deficits observed in hepcidin-resistant ferroportin *Fpn*^C326S^ mutant mice,^58^ and in hepcidin-deficient mice^59^^,^^60^; notably, the latter 2 models present the highest iron loading among all hemochromatosis models.

Together, all the data shows that we are still lacking clear delineation of underlying molecular mechanisms mediating iron-induced bone loss. This highlights the need for careful segregation between different modalities of iron overload when addressing when addressing iron’s effects on bone health.

### Limitations of the study

Despite this study using a robust translational model of iron overload and its skeletal impact, there are several limitations present that should be addressed.

First, the use of only female mice is limiting since there are known sex differences in the pathological progression of iron overload disorders in patients,^6^ and of bone status in mice.^61^ It would be important to inspect how the bone status in male mice is impacted in these models of iron overload.

Second, we started our treatment in relatively young mice (7-9 wk of age), when the bone mass is at its highest.^61^ Thus, our starting time points allowed investigation of the impact of chronic iron overload upon dynamic bone maturation; however, genetic iron overload manifests later in patients. Thus, it would be of importance to assess the impact of iron overload in aged mice, although certain bone parameters are significantly affected with age, making the analysis more challenging. However, assessing iron effects in mice with osteoporotic characteristics, such as those present in menopause/gonadectomy-induced bone loss, would be highly relevant for clinical settings and would induce an aged skeletal phenotype.^62^

Third, the endpoints of our 2 models are different, we selected an endpoint before 12 mo to investigate bone loss without the compounding factor of aging. However, while the endpoints of our treatments do not allow for a direct comparison of femoral Tb.N, for example, which decreases from 2 to 8 mo of age, certain bone parameters can be indirectly compared. For example, (1) L5 trabecular volume is maintained between the ages of 3-12 mo,^61^ (2) femoral and tibial cortical bone also show more stable composition over time and major bone parameters are conserved between 3 and 12 mo of age,^63^^,^^64^ and (3) BMD has a stable value until at least 20 mo of age.^65^

## Supplementary Material

Suppl_Figures_1-3_revision_ziaf118

SUPPLEMENTARY_FIGURE_LEGENDS_revision_without_track_changes_ziaf118

## Data Availability

The data that support the findings of this study are available from the corresponding author upon reasonable request.
