# Supplementary material for: Comparison between dietary, parenteral, and genetic iron overload on bone health reveals secondary iron overload as a driver of cortical bone loss and fracture risk in mice
Source: JBMR Plus. 2025 Jul 13;9(10):ziaf118. doi: 10.1093/jbmrpl/ziaf118 (PMC12418933; doi:10.1093/jbmrpl/ziaf118)

Supplementary Figure 1.

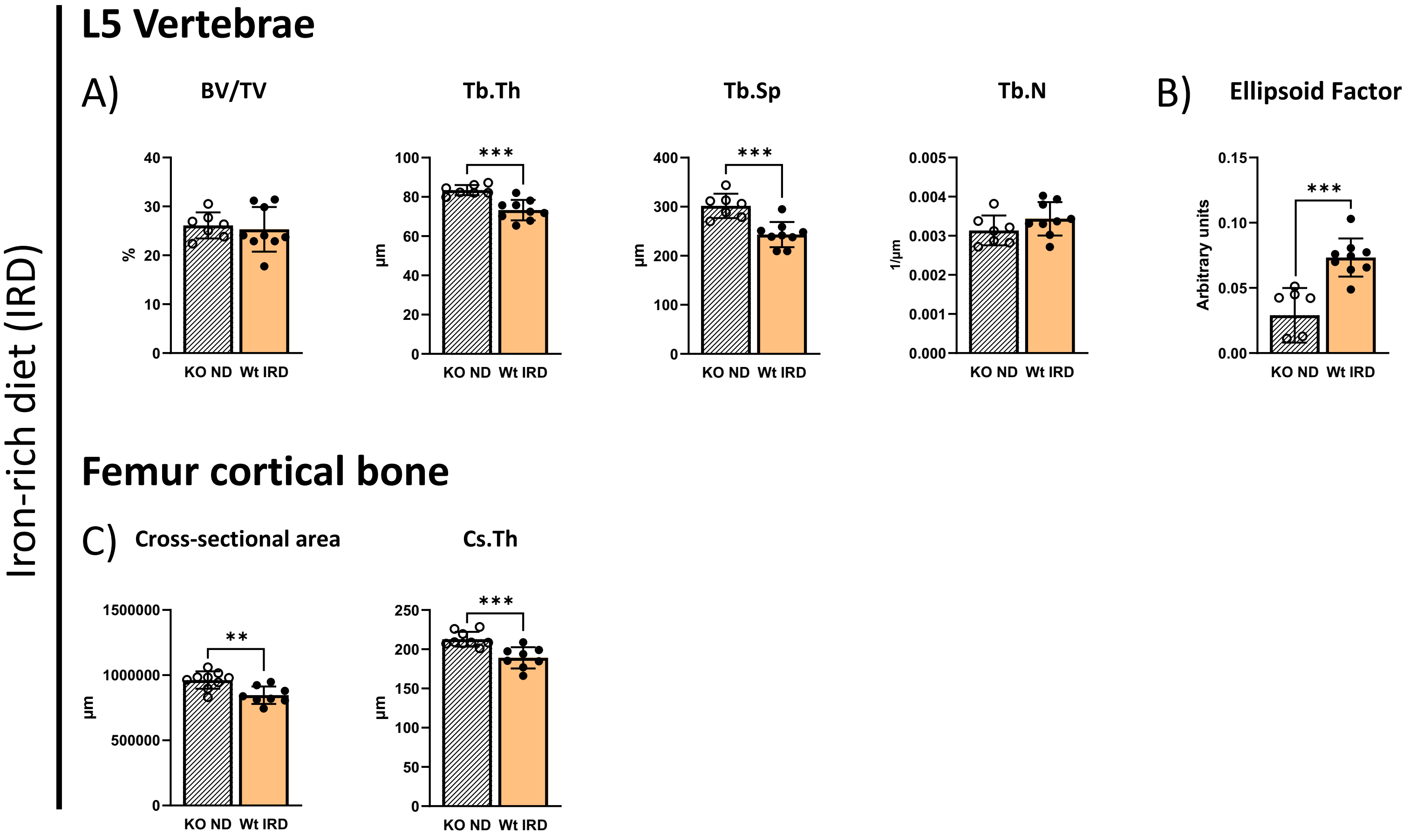

Supplementary Figure 2.

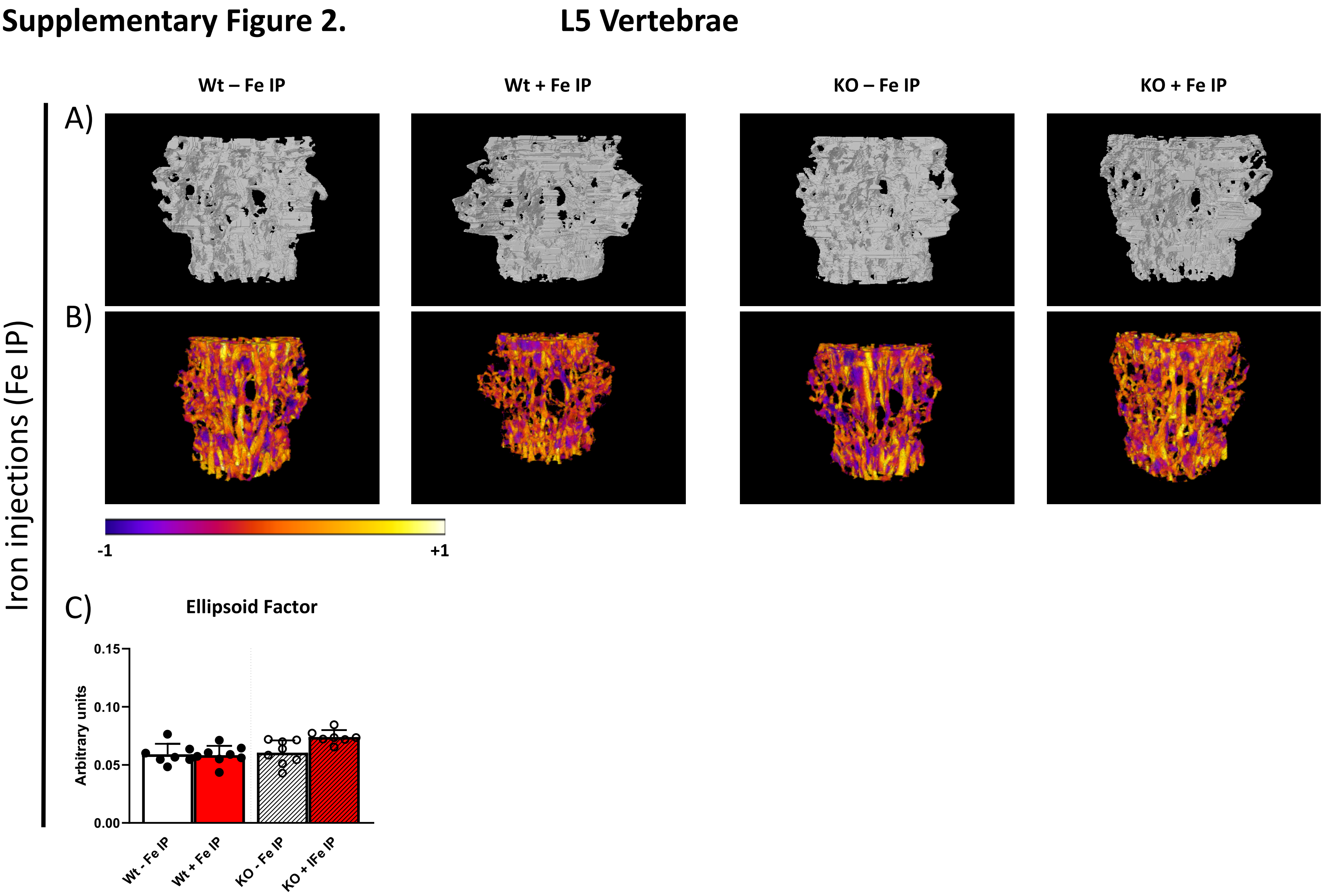

Supplementary Figure 3. **Femur trabecular bone**

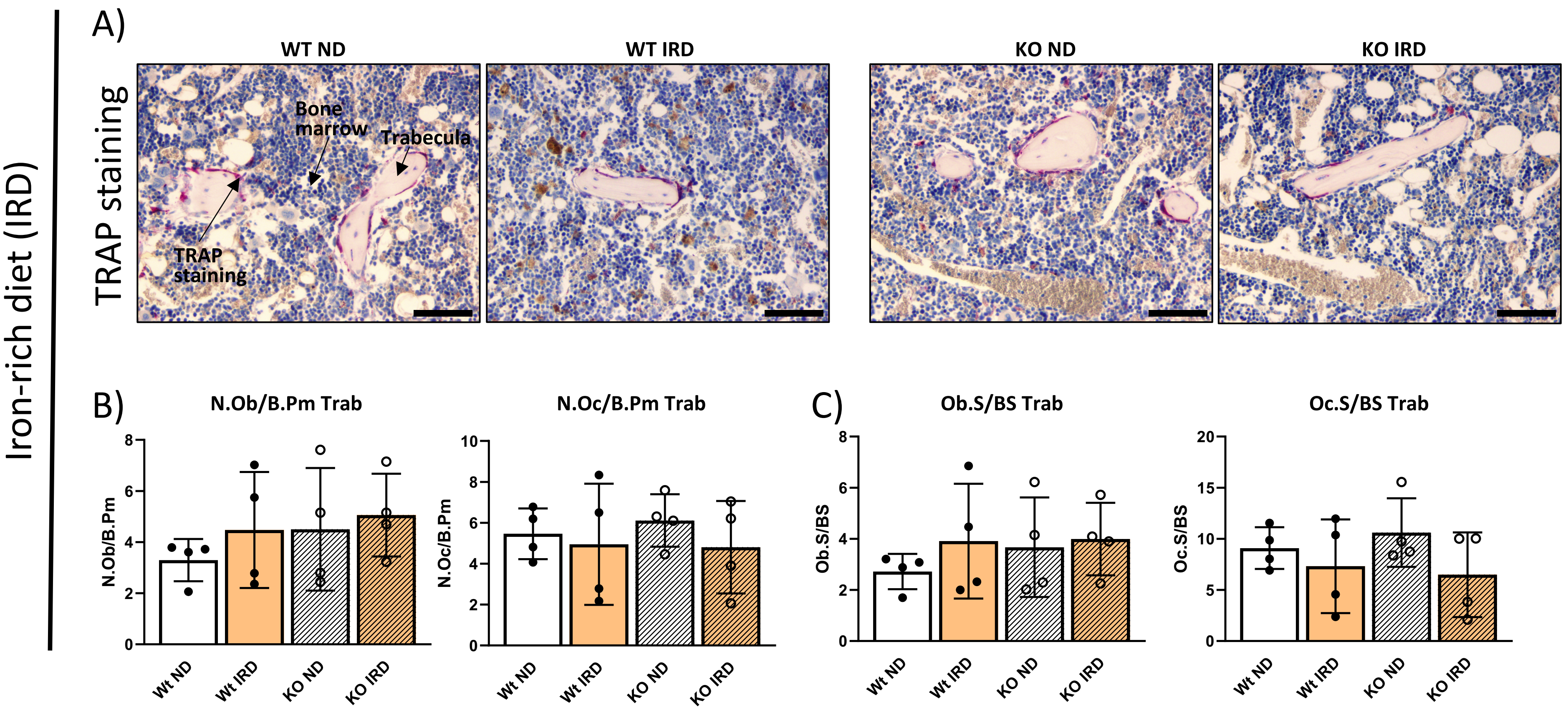

Supplement: Suppl_Figures_1-3_revision_ziaf118 [file suppl_figures_1-3_revision_ziaf118.pdf]
