# Supplementary material for: Comparison between dietary, parenteral, and genetic iron overload on bone health reveals secondary iron overload as a driver of cortical bone loss and fracture risk in mice
Source: JBMR Plus. 2025 Jul 13;9(10):ziaf118. doi: 10.1093/jbmrpl/ziaf118 (PMC12418933; doi:10.1093/jbmrpl/ziaf118)
Supplement: SUPPLEMENTARY_FIGURE_LEGENDS_revision_without_track_changes_ziaf118 [file supplementary_figure_legends_revision_without_track_changes_ziaf118.docx]

**SUPPLEMENTARY FIGURE LEGENDS**

**Supplementary Figure 1. Comparison between the effects of dietary and genetic iron overload on bone status**

(**A**) Trabecular bone parameters in the L5-vertebra of Wt mice on IRD and KO mice on standard diet (KO, ND) were assessed using μCT, displaying bone volume per total volume (BV/TV), trabecular thickness (Tb.Th), trabecular separation (Tb.Sp), and trabecular number (Tb.N). (**B**) Ellipsoid factor indicating trabecular plate-to-rod status was assessed on L5 vertebrae of Wt mice on IRD and compared to KO mice (KO, ND). (**C**) Cortical bone parameters in the femur of Wt mice on IRD and KO mice (KO, ND) were assessed using μCT, displaying cross-sectional bone area and cross-sectional thickness (Cs.Th). The data is presented as the mean ± SD, with each symbol representing an individual animal. *p < 0.05, **p < 0.01, ***p < 0.001.

**Supplementary Figure 2. Effects of parenteral iron overload on the spine**

(**A**) Representative 3D rendering of the L5 trabeculae of Wt and KO mice on Fe IP and controls in the region of interest. (**B**) Ellipsoid factor heat maps were generated from the L5 vertebrae trabecular of mice upon Fe IP and controls, with purple indicating a plate-like trabecular structure and yellow a rod-like trabecular structure. (**C**) Ellipsoid factor indicating trabecular plate-to-rod status was assessed on L5 vertebrae of mice subjected to Fe IP and controls. The data is presented as the mean ± SD, with each symbol representing an individual animal. *p < 0.05, **p < 0.01, ***p < 0.001.

**Supplementary Figure 3. Effects of dietary iron overload on bone cells**

(**A**) Tartrate-resistant acid phosphatase (TRAP) staining in the femur of mice on IRD and controls. Scale bar indicates 50μm. Arrows indicate trabeculae, bone marrow and TRAP staining. (**B-C)** Bone histomorphometry showing the number of osteoblasts/osteoclast relative to the bone parameter (N.Ob/B.Pm, N.Oc/B.Pm) and the surface of osteoblasts/osteoclast relative to the bone surface (Ob.S/B.S, Oc.S/B.S). The data is presented as the mean ± SD, with each symbol representing an individual animal. *p < 0.05, **p < 0.01, ***p < 0.001.
